# Ethical values supporting the disclosure of incidental and secondary findings in clinical genomic testing: a qualitative study

**DOI:** 10.1186/s12910-020-0452-0

**Published:** 2020-01-30

**Authors:** Marlies Saelaert, Heidi Mertes, Tania Moerenhout, Elfride De Baere, Ignaas Devisch

**Affiliations:** 1 Department of Public Health and Primary Care, Philosophy of Medicine and Ethics Research Group, Ghent University, Corneel Heymanslaan 10 – Building 6K3, 9000 Ghent, Belgium; 2 Department of Philosophy and Moral Sciences, Bioethics Institute Ghent, Ghent University, Ghent, Belgium; 3 Department of Philosophy and Moral Sciences, Ghent University, Ghent, Belgium; 4 Center for Medical Genetics Ghent (CMGG), Ghent University and Ghent University Hospital, Ghent, Belgium

**Keywords:** Patient autonomy, Professional beneficence, Soft paternalism, Distributive justice, Clinical genomic testing, Incidental findings, Secondary findings, Qualitative research

## Abstract

**Background:**

Incidental findings (IFs) and secondary findings (SFs), being results that are unrelated to the diagnostic question, are the subject of an important debate in the practice of clinical genomic medicine. Arguments for reporting these results or not doing so typically relate to the principles of autonomy, non-maleficence and beneficence. However, these principles frequently conflict and are insufficient by themselves to come to a conclusion. This study investigates empirically how ethical principles are considered when actually reporting IFs or SFs and how value conflicts are weighed.

**Methods:**

A qualitative focus group study has been undertaken, including a multidisciplinary group of professionals from Belgian centres for medical genetics. The data were analysed thematically.

**Results:**

All eight Belgian centres participated in this study. Ethical values were frequently referred to for disclosure policies on IFs and SFs. Participants invoked respect for patient autonomy to support the disclosure of IFs and opt-out options for IFs and SFs, non-maleficence for the professional delineation of reportable IFs and opt-out options for IFs and SFs and (the particular scope of) beneficence for the mandatory reporting of actionable IFs, the delineation of reportable IFs and a current decline of actively pursued SFs. Professional assumptions about patients’ genetic literacy were an important factor in the weighing of values.

**Conclusions:**

In line with the traditional bioethical discourse, the mandatory reporting of actionable IFs might be interpreted as a “technological, soft paternalism”. Restricting patients’ choices might be acceptable, but then its motives should be valid and its beneficent outcomes highly plausible. Hence, the presuppositions of technological, soft paternalism - patients’ inability to make informed decisions, normative rationality, the efficacy of beneficent outcomes and the delineated spectrum of beneficence - should be approached critically. Moreover, distributive justice should be considered an important value in the delineation of the current scope of the ethical debate on IFs and SFs.

This study of guiding values may stimulate the debate on the ethical grounds for a solid policy on IFs and SFs internationally.

## Background

In clinical exome sequencing (ES), variants in diagnostically unrelated but known disease genes can be unintentionally revealed or actively pursued as, respectively, incidental findings (IFs) and secondary findings (SFs) [1–3]. Incidental and secondary findings are the subject of various reporting guidelines and policy documents, for instance in Europe, the US and Canada [1–7]. Ethical arguments, especially concerning autonomy, non-maleficence and beneficence, have been frequently cited for reporting these results or not doing so [8, 9]. The study presented in this article set out to investigate empirically how professionals consider these and potentially other values in actual practice regarding IFs and SFs in clinical ES.

International healthcare conventions have formalised respect for patient autonomy in the right to receive personal and complete health information (including informed consent before a medical treatment), as well as in the right to decline medical information, treatment and intervention [10–14].

In line with these rights, non-disclosure of clinically relevant information has been ethically rejected and a patient’s right to be informed about (specific) IFs has been acknowledged [8, 15]. However, whether this right also installs the professional duty to deliberately pursue additional findings as SFs, is contested. The American College of Medical Genetics and Genomics (ACMG) advocates the opportunistic screening of a well-defined list of genes which are clinically significant, highly penetrant (i.e. with a high probability that the pathogenic variant will express the associated condition) and medically actionable (i.e. allowing medical prevention or treatment) [1]. According to the ACMG, this pursuit of SFs is the most effective realisation of patients’ (family-wide) wellbeing and hence of beneficence, a professional commitment which takes a prominent place in, for example, the Declaration of Geneva [1, 5, 16]. Conversely, the European Society of Human Genetics (ESHG), EuroGentest and the Canadian College of Medical Geneticists (CCMG) are more cautious with diagnostically unrelated results; they recommend a minimisation of IFs and they explicitly discourage or seem not to support the active pursuit of SFs [2, 4, 6]. Arguments for this cautiousness are the possibility of physical and/or emotional harm (by overwhelming patients with unnecessary or harmful tests, diagnoses or interventions) and hence the professional duty of non-maleficence [3, 8]. When IFs are unintentionally identified, these results should only be disclosed if they are highly significant, highly penetrant and medically actionable [17–19].

With respect to patients’ right not to know, there is a consensus that explicit patient consent is required for the screening and reporting of SFs [3, 20–22]. Since its updated recommendations, the ACMG also agrees with a possible patient opt-out for SFs [23]. Respecting patients’ choice on disclosure is motivated by the idea that information about genetic predispositions cannot be imposed because of its possible psychological, family and social impact [24]. This indicates that the right not to know is supported not only by the value of patient autonomy but fundamentally grounded in the interest of not being psychologically harmed and hence in the professional duty of non-maleficence [24]. Nonetheless, and despite the consensus on an opt-out possibility for SFs, the opt-out of IFs has been debated more intensively. Whereas some professional bodies, such as the CCMG, strongly uphold patients’ right not to know, the ESHG and EuroGentest recommend that the final decision on serious and actionable IFs should be made by professionals [2, 4, 6]. Consequently, the professional responsibilities to warn, rescue and benefit patients might outweigh the patient’s right not to know [3].

The weighing of prima facie values such as patient autonomy and professional beneficence is a classic challenge in bioethics [25] and the debate on IFs and SFs turns out to be a prime example of it. Consequently, opposing policies are advocated and many questions are still unanswered. Under which conditions should a patient’s wish to opt out of IFs be respected? If this right is not absolute, how (for instance based on which criteria or values) can professionals justify their decision to report these results without patient consent? Should SFs be deliberately pursued as a realisation of the professional duty of care and the patient’s right to be informed? And more fundamentally: are autonomy, non-maleficence and beneficence actually guiding principles in professionals’ decisions about disclosing IFs and SFs? Or is there a gap between theoretical ethical concerns and practice [26]?

The question whether and how professionals consider these and potentially other values as guiding notions in the reporting of IFs and SFs in a context of diagnostic ES in adults, is the focus of this article.

## Methods

A qualitative study was organised in the eight Belgian centres for medical genetics (CMGs) to achieve an in-depth understanding of professionals’ perspective on IFs and SFs. Since the aim of this study was not to determine role-specific or individual views but the integrated perspective of a group of professionals who collaborate in a CMG and might decide on the disclosure of IFs or SFs after inter-professional deliberation, focus groups were chosen over individual interviews [27]. Aiming for an active debate and open conversation between colleagues, one focus group in every CMG was pursued [27]. A purposive sampling approach was used in every CMG to recruit a multidisciplinary and representative group of professionals who are experienced with clinical ES, including both clinical geneticists and clinical laboratory geneticists and possibly other professionals such as genetic counsellors, bio-informaticians or nurses [28]. Through a presentation at the Belgian College of Medical Genetics (a federal body for the quality of healthcare in medical genetics), representatives of all CMGs were informed about our study and contact information was collected from one or several professionals (usually including the head of department) at every CMG. Subsequently, a contact at every CMG was approached by email or telephone by MS to provide additional information about the focus groups and to request participation. At the contact’s request, a preliminary consultation was organised at several CMGs to thoroughly clarify the design and aim of the focus group. If the contact agreed to participate, (s)he or another professional at the CMG contacted eligible colleagues and assembled a representative group of people. To counter last-minute cancellations, contacts were requested to assemble a group of about twelve persons. When participants had been recruited, the contact suggested a time which suited most of the CMG’s professionals.

Focus groups were conducted between November 2016 and December 2017 in a room at the CMG or associated hospital and lasted between 67 and 117 min. The first author moderated all focus groups in Dutch or English and participants replied in English, French or Dutch. In seven out of eight focus groups, an observer was present and took field notes. Informed consent was obtained from all participants.

A semi-structured interview guide, created after a literature review and including open-ended questions, was used for all focus groups (Table 1). At the beginning of every focus group, the focus on IFs and SFs in clinical ES for monogenic diseases, excluding preconception, prenatal, screening and research contexts, was emphasised.

**Table 1:** Examples of interview questions

How do you define IFs in clinical ES?	
What kind of IFs do you report, firstly, from the laboratory to the clinician and, secondly, from the clinician to the patient?	
What do you think about the intentional search for SFs?	
What kind of policy regarding IFs and SFs would you like to develop?	
What difficulties do you experience in your practice and (future) policy regarding IFs and SFs?	
How is the possibility of IFs addressed during genetic counselling?	
What might affect a patient’s interest in IFs and SFs?	
What is your policy regarding a patient’s possibility to opt out of IFs?	
How would you define a patient’s role in the context of IFs and SFs? How does this role relate to your professional role?	
What impact might a reported IF or SF have on patients?	
To what extent do you consider a personalised policy concerning IFs and SFs appropriate and feasible?	

Abbreviations used: *IF(s)* Incidental finding(s); *SF(s)* Secondary finding(s); *ES* Exome sequencing

Focus groups were audio-recorded and transcribed verbatim and data were saved on a password-protected server. The data were analysed thematically [29]. The inductive and iterative analysis process was supported by use of the software program NVivo 12 and reflective ideas were stored in memos. To assert the trustworthiness of the data collection, analysis and reporting, an extensive procedure was elaborated, which combined peer debriefing and a systematic audit trail [30]. TM conducted a secondary analysis of a substantial subset of the data. Consequently, TM and MS discussed transcripts and initial code schemes, as well as theme names and definitions. Thematic structures and draft reports were reviewed by the multidisciplinary group of all authors until consensus was reached between them all. Finally, illustrative quotes were selected and, if originally in Dutch or French, translated by MS and TM.

This article adheres to the COREQ-guidelines for reporting qualitative research [31].

## Results

All eight Belgian CMGs participated in this study, with a total number of 68 participating professionals (Table 2).

**Table 2:** Focus group participants

	FG1	FG2	FG3	FG4	FG5	FG6	FG7	FG8	Total
Function participants									
Clinical geneticists	3	3	4	5	3	3	2	2	25
Clinical laboratory geneticists	3	3	4	2	4	2	2	6	26
Genetic counsellors/Psychologists		4	1	2	1	1	2		11
Others (Bio-informaticians, Bioethicists, MD trainees)		1		1			3	1	6
Total	6	11	9	10	8	6	9	9	68

Abbreviations used: *FG* Focus group

Even though participants were not explicitly asked for principles that supported their reporting practices regarding IFs and SFs, professionals frequently referred to ethical values including autonomy, non-maleficence and beneficence, and these concepts emerged from the data as a specific theme. More generally, the identified themes regarded: (i) current and general practice in clinical genetic testing, (ii) the position of genetics in medicine and society, (iii) criteria for reporting IFs, (iv) impact of IFs and SFs, (v) policy guidelines for genetic practice, (vi) guiding values and principles. This article specifically addresses the sixth theme in a context of clinical ES in adults.

### Patient autonomy and the right to know

Based on a patient’s right to receive relevant information, all participants agreed on patients’ right to be informed about some IFs. Therefore, and in the interests of a just policy, all patients should have equal opportunities to receive relevant IFs, independent of the testing techniques used. On the other hand, all Belgian CMGs only reported IFs and did not actively pursue SFs (cf. infra).

Due to all CMGs’ current clinical practice of ES-based but filtered panel testing in which a set of known disease-associated genes is analysed, the chance of identifying an IF is not zero, but it is rather small and professionals from all CMGs reported limited experience with IFs in clinical ES. As the significance of ever more genes becomes known and used panels contain an increasing number of genes or when testing techniques evolve (and, for instance, include whole exome or whole genome analysis or genome-based panels), it is assumed that the number of IFs will increase.

Several professionals advised that, because of the current use of panels and to avoid unrealistic experiences, patients should be informed that, at this time, not all diagnostically unrelated health risks will be identified. However, it was assumed that people’s requests for genomic information would increase over time. Consequently, many professionals stressed that, when whole exome or whole genome sequencing becomes basic clinical testing and when it becomes technically feasible to comply with people’s growing requests for information, the general population should have a better understanding of genomics and its possible consequences and limits. Unrealistic expectations and genetic determinism should be avoided and people should realise that genetics cannot explain or predict all (health) concerns.

*“We have the impression that people go to the geneticist as if they were going to a fortune teller with a crystal ball. [After the consultation] they say “well, this is my future”. While we say “I can’t tell you anything with a genetic test. I don’t know if you have cancer and I don’t know if it will come. I can’t tell you anything.”* (P5, FG5).

In line with the need for better informed citizens, the importance of genetic counselling was unanimously emphasised. Counselling should inform patients about the possible outcomes of the test, including IFs, in comprehensive, non-technical terms. Finally, new ways of counselling were suggested, such as collective counselling sessions where general genetic concepts or frequent conditions could be explained.

### Patient autonomy and the right not to know

Participants described how ES-based panel testing (as a selection of analysed genes) generally avoids the identification of IFs, which supports the opportunity to respect a patient’s wish not to know diagnostically unrelated results. Participants suggested two motives for a patient’s preference to opt out of IFs: emotional distress and diagnostic focus. Firstly, the prospect of additional genetic information might engender anxiety and patients might not want to or might not feel psychologically able to deal with this information. One professional explicitly associated a preference not to know with emotional motives, whereas a preference to know was associated with rational motives, for example regarding therapeutic options. Moreover, the inherent degree of uncertainty in IFs and in genetic results in general (because of incomplete penetrance or variable expression, i.e. the variable manner in which a condition is manifested) might engender feelings of doubt instead of knowledge and assurance. Secondly, patients were described as focussed on receiving a diagnosis for their symptomatic condition and hence they considered IFs and SFs less important side notes. This argument was also stated by a professional of a CMG without an opt-out possibility for actionable IFs, but it was in line with some professionals’ doubts regarding the centre’s current practice.

Besides these two patient motives, some professionals explicitly referred to the fundamental value of patient autonomy and the included right not to know as arguments for unconditionally respecting a patient’s preference. Half of the Belgian CMGs always allowed an opt-out from IFs, including actionable results which were specified as findings for which medical treatment or preventive screening are available. Participants argued that patients cannot be forced to receive unwanted information and that a preference “to stay in denial” should always be respected. The professional duty to avoid psychological harm and emotional distress, potentially caused by IFs, also favoured the possibility of an opt-out.

*“When the patient says “No, I don’t want to have any other result than what we are looking for”, then I think you should not report it. […] Therefore, I think, genetic counselling is very valuable and you have to do everything to respect your patient. I think that’s the most important thing. It’s not up to us to decide what to report and what not […].”* (P7, FG3).

Some professionals suggested that when a patient opts out, IFs might still be reported from the laboratory to the clinician, so clinicians can be attentive for early symptoms during follow-up consultations. It also allows for reporting the IF at a more suitable moment or when the patient asks about it later. Other professionals, however, suggested that declined IFs should be masked in the laboratory report, so situations where the clinician knows but cannot disclose relevant information to the patient are avoided.

Finally, participants discussed the possibility of a selective opt-out from specific (categories of) IFs and professionals at two CMGs (both allowing an opt-out of actionable IFs) would support this practice as soon as IFs can be accurately categorised. However, explaining these categories might become too complex, especially when the number of reportable IFs increases. Professionals at two CMGs without an opt-out possibility already felt that this practice was too complicated. It would increase the professional workload and patients were considered unable to make these stratified choices.

### Genetic literacy, patient autonomy and professional beneficence

In all CMGs, a major challenge in clinical ES was discussed, being patients’ inability to fully understand the meaning and consequences of IFs. Participants mentioned several reasons for people’s limited genetic literacy and inadequate understanding. Firstly, genetic information might be conceptually new, complex, extensive and overwhelming. Secondly, and in contrast to standard medical tests, IFs are usually not expressed in related symptoms and do not reveal an “instant reality”. This presymptomatic risk assessment with a delayed relevance and possibly lifelong impact might be difficult to interpret or to use as grounds for decisions. Thirdly, conditions’ (incomplete or age-dependent) penetrance might be difficult to understand, especially in unexpressed IFs, and people might not be used to thinking about risks or chances. Finally, genetic tests might be prescribed by non-geneticists. Combined with a lack of time for adequate pre- or post-test counselling, these professionals’ limited experience with genetic medicine might result in an incomplete transfer of information and “uninformed” consent from patients.

Some professionals did not believe that patients’ lack of understanding could be resolved within the timeframe of a counselling session and hence they did not believe it was possible for patients to make informed decisions about IFs. Consequently, three Belgian CMGs did not allow an opt-out from actionable IFs. One professional mentioned that opting out can provide temporary psychological relief but eliminates neither the medical risk nor the psychological distress in the long run. Refusing an actionable IF is only a short-term remedy that postpones distress from the time of knowing to the time of expression. Professionals at CMGs without an opt-out possibility feared that patients did not fully understand the potential consequences of opting out, for instance the future benefit that might be declined. Patients might regret it when, later, a medically actionable condition (for example breast cancer) manifested and they might blame professionals for non-disclosure of this risk.

*“[…] I think the majority of them [patients], 99% of them, do not know what they are agreeing to or what they are not agreeing to, when they say “I don’t want to know or I do want to know.””* (P8, FG3).

In addition to the argument concerning patients’ genetic literacy, several professionals expressed a feeling of responsibility towards patients. They would experience it as psychologically unbearable and inappropriate to observe but not report a health risk of a possibly preventable condition. Hence for some professionals, this perceived duty of beneficence outweighed the value of patient autonomy and supported the absence of an opt-out possibility for actionable IFs. Professionals of all CMGs debated the interaction between professional beneficence and patient autonomy and in one CMG, this interaction in the case of a potential opt-out was the main point of discussion. Whereas most professionals at this centre advocated a right not to know actionable IFs, one professional upheld the idea of overruling an opt-out choice and nevertheless reporting a medically actionable IF. Arguments for this infringement of a patient’s choice were the belief that the consequences of an unreported IF could be more severe than those of denying a patient’s preference and, again, the belief that patients do not understand the possible consequences of their own opt-out choice.

*“For something like a BRCA1-deletion […], I would not accept an opt-out and I would inform the patient anyway, saying “well, the consequences are so big, so important, [that] I consider it medically more important that you know, than actually to respect your autonomy as a patient.”* (P8, FG3).

In defence of a mandatory disclosure of actionable IFs, few patients were said to dispute this policy, which suggested patients’ trust in the professional practice. It also supported a participant’s statement that a mandatory disclosure should not be qualified as a paternalistic act but as acting in line with patients’ interests and with their need for guidance along the diagnostic quest.

Even though adequate understanding for autonomous decision-making was not considered possible by everyone, genetic counselling was generally estimated essential and effective to prepare patients for a potential (mandatory) disclosure of IFs.

*“You can compare it to a pregnancy ultrasound. Why do families or mothers want a pregnancy ultrasound? To hear that everything is okay. Very few people think about the possibility of bad news and how to deal with it. And I think, when people enter the [genetic] centre, this is one of our essential tasks. […]. So if you incidentally find a [variant in a] Lynch-gene [included in a breast cancer panel], you can say something like “look, this is not the answer, but we have found something else of importance.” It is an essential part of our job that, at that time, this [information] is not completely new to patients.”* (P7, FG2).

Professionals also acknowledged individual variance in the capacity to understand or emotionally bear genetic information. Personalising the policy on IFs (for example by offering some patients more options) was, however, regarded as undesirable because it violates the value of equality and stimulates favouritism and a dual healthcare system. Moreover, offering personalised options entails the difficulty for professionals of estimating a patient’s situation and capacities correctly.

It should be noted that at two of three CMGs without an opt-out possibility, professionals did not only rely on ethical arguments but also referred to procedures prescribed by the local ethics committee. Participants mentioned that the ethics committee did not allow an opt-out because it assigned professionals the responsibility of reporting available and useful information and because it considered patients incapable of informed decision-making in the context of clinical ES. Remarkably, some professionals at these CMGs said that if the ethics committee were updated on recent evolutions in clinical ES, a re-evaluation of the opt-out policy might be possible. However, no in-house consensus was reached on this idea.

As a final remark, several professionals stated that, depending on the patient’s best interests (including a correct diagnosis), a patient’s genetic illiteracy is no reason to dismiss a genetic test *in se*. Even after counselling, numerous patients will not fully realise the meaning and possible consequences of ES (including the possibility of IFs). However, the test would usually be performed anyway, as this was considered to benefit a patient’s care.

*“I try my best to explain it, but when I notice, at a certain moment, that it [a patient’s understanding] stops, but they want another child, then I think, also for the best interest of the patient, let’s just start another test. […] Doing nothing, because you think they have not fully understood, while you think it is in their interest to continue, well, then I think, as a clinician: “What would be the best choice of several options?”* (P10, FG4).

### The scope of ethical values

The last theme applies to the scope of ethical values and their application in practice. This theme was clearly observed in three particular issues concerning the disclosure of diagnostically unrelated findings.

Firstly, despite patients’ right to be informed about IFs, this right was limited by professionals’ duty of non-maleficence. There was a consensus among professionals at all CMGs to specifically delineate the scope of reportable IFs. Most participants advocated a restriction of reportable IFs to class 5 and class 4 (pathogenic and likely pathogenic) variants in medically actionable genes, but several professionals indicated that this spectrum might change when scientific knowledge increases or societal interests and taboos change. Only one professional mentioned that the professional delineation of reportable IFs could be perceived as “rather paternalistic”. Conversely, at more than half of the CMGs, participants stated that their professional expertise should compensate for patients’ genetic illiteracy and that it is their professional responsibility to decide which findings are comprehensible for lay people and hence relevant to report. Moreover, reporting “ambiguous” or “nonsensical” data (for instance class 3 variants of uncertain significance (VUS) or medically non-actionable IFs) might result in harmful interventions, (unnecessary) fear or false feelings of certainty, and professionals assigned themselves the duty of avoiding these possible harms.

*“Professional bodies have decided that it’s about the actionables. Hence only cancer and cardiac conditions have been included [in lists of reportable results]. I think, if you include more, it will become very stratified and one might wonder whether patients still understand what they are signing up for.”* (P11, FG2).

*“Different systems are being used and some [genetic] centres say “let’s offer different choices to the patient” and this can go very far. Patients can choose not only whether they want to receive [additional results] or not but also which [additional results] they want to receive. […] I have even seen an [informed consent] form which asked whether you want to receive variants of non-significance or not. So where the idea is something like “in the laboratory, we can’t figure it out, so let’s leave it up to the patient.”* (P1, FG2).

In the delineation of reportable IFs, some professionals considered the health of patients’ family members as included in the duty of beneficence and hence they supported the disclosure of IFs regarding a carrier status of a recessive condition.

*“When patients find out, during a later pregnancy, that their child has Duchenne [muscular dystrophy] although we have seen it in their older daughter… You don’t want this to happen. That’s why we don’t work with an opt-out, to avoid this kind of thing.”* (P2, FG2).

Secondly, the scope of professional beneficence was sometimes delineated by the spectrum of the “clinical gaze”. Some professionals characterised medical responsibilities as not strictly limited to the diagnostic question, which resulted in a more extended perspective of a patient’s health. Mainly professionals at two CMGs without an opt-out possibility for actionable IFs advocated this holistic clinical gaze.

*“It also creates a responsibility, I think, when a patient consults you for a condition and there is also something else in the family which might be important, that you have to keep this in mind and do something about it. We have had such a discussion, about someone who consulted us for cancer while there was also a history of aneurysms in the family. This was not followed up and the patient died of an aorta aneurysm. Afterwards, it was discussed whether it was the counsellor’s responsibility to follow up on this.”* (P9, FG4).

Other participants, however, defined their fundamental responsibility as more restricted. Patients were characterised as diagnostically focussed and aiming for a specific answer to a particular question. To address this request and to answer patients’ questions most efficiently, professionals should adopt this diagnostically focussed clinical gaze. Hence, these professionals supported the use of specific, demand-driven tests that minimise the chance of additional findings.

*“I think that we, clinicians, should try to avoid finding IFs as much as possible and so we should use as many filters as possible to avoid them.”* (P3, FG5).

Finally, and associated with the scope of beneficence, several participants mentioned and showed enthusiasm for a future practice of SFs (described as a practice of opportunistic screening with a presymptomatic risk disclosure). This possibility was mentioned by professionals at both CMGs with and without an opt-out possibility for IFs and it was presented as a practice that could meet patients’ increasing demand for genomic information and a practice that could achieve a higher level of care. Some professionals even feared that denying a practice of SFs could someday be labelled a medical error. However, there was a consensus among professionals at all Belgian CMGs not to routinely pursue SFs yet and to consider this practice as currently falling outside the scope of beneficence. Various underlying reasons were mentioned for this limited scope. Firstly, a practice of SFs might be a disproportionate investment of limited budgetary, logistical, human and technical resources. Combined with a lack of specific guidelines, this could result in less valid and potentially harmful results and it would disadvantage a CMG’s workflow and lengthen the waiting time for diagnostic results. Secondly, and partly due to lay people’s genetic illiteracy, society was not considered ready for a routine practice of SFs. Finally, it was suggested that patients fundamentally do not want to receive additional results (whether IFs or SFs) because of the intrinsic “bad news” they include. Even though IFs or SFs might be useful, no patient wants to be confronted with additional health risks. Therefore, there was an agreement among professionals on patients’ future right to opt out of SFs. This right was also stressed by professionals of CMGs without an opt-out possibility for actionable IFs.

*“You cannot offer a kind of package deal and say “we are going to do this test and you are also obliged to accept these SFs […].” That is something you can’t do, it would be unethical.”* (P1, FG7).

The fundamental restraint towards additional “bad news” did not only apply to patients. At two CMGs, participants expressed the professional feeling of emotional distress caused by IFs. Neither patients nor professionals are looking for IFs and a confrontation with these findings is unpleasant to both parties. Hence reporting IFs was characterised as “a dirty job” that still needs to be done; finding the balance between autonomy, beneficence and non-maleficence was experienced as “mental gymnastics” for professionals.

*“Most people don’t really want to know [this information] anyway, but I think, if you find it, they should know. But it’s… I try not to get in that situation, if possible […] If I ask for a cardiomyopathy, I don’t want to find BRCA mutations, I don’t want to find a mental retardation mutation! […] as a medical person, as a doctor, I feel that I have to do it, but still, if I were the patient, I wouldn’t be pleased to find out. I would rather know, but I wouldn’t be pleased about it.”* (P8, FG3).

## Discussion

Professionals at Belgian CMGs frequently justified their centre’s practice and policy regarding IFs and SFs by ethical principles. As a consequence of the use of ES-based panel testing and a limited experience with IFs in clinical ES, it should be acknowledged that these justifications might not only consider actual practices but also preferable future policies.

In line with international scholarly literature, professionals frequently referred to principles of autonomy, non-maleficence and beneficence [8]. The disclosure of IFs was supported by respect for patient autonomy, the professional delineation of reportable IFs was supported by non-maleficence and the spectrum of beneficence, and the decision not to actively pursue SFs was supported by the currently limited scope of beneficence. The possibility of opting out of actionable IFs was the most discussed element during the focus groups and various ethical values regarding this practice were weighed up. Allowing an opt-out was justified by the values of autonomy (respecting a preference not to know) and non-maleficence (not inflicting psychological or medical harm), whereas not allowing an opt-out was mainly justified by the principle of beneficence (preventing future medical harm as a duty of care). The weighting of these values was strongly influenced by professional ideas about patients’ genetic literacy, their (inadequate) understanding of ES and IFs and their ability to make informed and autonomous decisions. These assumptions affected professionals’ final choice regarding an opt-out possibility and resulted in mandatory reporting of actionable IFs at some Belgian CMGs. Mandatorily reporting of IFs, irrespective of patients’ preferences, might sound contestable in current, patient-centred ideologies, but it is supported by recommendations by the ESHG and EuroGentest, which advocate a professional final decision regarding the disclosure of serious and actionable IFs [2, 4]. Conversely, the policy of half of the Belgian CMGs that allow an opt-out from IFs is supported by Vears et al.’s points to consider for laboratories and by the CCMG position statement which states that “competent adults should be given the option prior to testing to receive (or not receive) incidental findings unrelated to the primary test indication.” [6, 7].

If the reporting of IFs occurs against a patient’s consent, this disclosure might be conceptualised as medical paternalism, i.e. interference in a patient’s autonomy without this patient’s consent, but only because the medical professional is genuinely concerned about patients’ health and wellbeing and thinks that his/her interference will benefit the patient [32–35]. Since the professional’s action consists of an epistemic intervention - the disclosure of medical information which is considered useful - the mandatory reporting of actionable IFs can also be labelled as “epistemic paternalism” [36, 37].

In line with traditional bioethical discourse, the mandatory disclosure of actionable IFs at three Belgian CMGs would be considered soft paternalism because patients are assumed to lack the genetic literacy to fully understand the consequences and impact of ES and IFs and hence, in this context, they are unable to make informed, autonomous decisions [25, 38, 39]. Soft paternalism is not uncontested but accepted by many as a common medical intervention and is, on occasion, also preferred by patients themselves [25, 40]. In addition to the previous conceptualisation, we will refer to the mandatory reporting of actionable IFs as *technological* soft paternalism. In comparison to other medical information that patients might understand, ES technology and the abundant and complex results it might generate (including IFs) are, also after standard pre-test counselling, considered very complex for the average patient [34, 41]. Hence the medical technology used is the specific and context-dependent cause of patients’ inadequate understanding and inability to make autonomous decisions, and it is the underlying technological justification of soft paternalism.

In summary, the technological, soft paternalism regarding actionable IFs can be characterised as the professional decision, motivated by patients’ best interests, to disclose actionable IFs because patients lack the genetic literacy to understand the technology of ES and its complex results and hence are, in this context, incapable of autonomous decisions.

Despite the technological, soft paternalism’s grounds in undeniably complex medical information and its benevolent focus on patients’ wellbeing, some remarks can be made regarding its justification and efficacy in the specific context of clinical ES and actionable IFs.

Firstly, as the soft paternalism is grounded in the specific context of ES technology and its complex results, the mandatory reporting of actionable IFs might be considered a modus of “procedural paternalism”: only in the specific context of genetic testing by means of ES technology and at the specific time of diagnostic testing are patients incompetent and non-autonomous and hence professionals are authorised to decide on the disclosure of results without patients’ consent [39], However, it is hard to claim that, even in this specific technological context, every patient lacks the genetic literacy to understand complex ES results, and hence is incompetent to decide autonomously about the disclosure of these results. It seems that exceptions to technological, soft and procedural paternalism should be allowed but it is unclear how these exceptions are compatible with the principle of justice. These reflections were also expressed by professionals from some Belgian CMGs and in participants’ restraint concerning more personalised choices regarding IFs because of their possible violation of the justice principle. Technological, soft paternalism could also be considered a type of “endangerment paternalism”, where actions are generally subjected to paternalistic actions because of the risk that at least *some* people are incompetent [39]. However, this would imply that some patients are limited in their actions without actual proof of their inadequate understanding, turning the paternalistic intervention into hard paternalism towards autonomous persons [39]. Moreover, autonomous decisions about IFs might not require a *full* and technological understanding of ES and instead, autonomy and genetic literacy might be considered a continuum. Rather than an absolute ideal, autonomy can be considered a threshold concept where patients have a *sufficient* understanding and are *sufficiently* competent and autonomous [36, 39]. It is also likely that this understanding should not focus on the technology of ES, but rather on comprehensible and practical consequences of test results, an idea which was, along with the suggestion about new ways of counselling, also raised by Belgian professionals [3, 42, 43]. The possibility or at least the pursuit of a *sufficiently* informed and autonomous patient does, however, not deny professionals’ more profound understanding of ES and genomic results, and this expert epistemic position applies generally in medicine [25]. Moreover, the complexity of ES results is generally acknowledged and literature has shown that the genetic literacy of the general population is rather limited [44, 45]. Hence doubts about patients’ ability to make informed decisions about IFs, even after pre-test counselling, are not uniquely Belgian concerns [17, 46]. In conclusion, rather than aiming for a fully informed patient decision-making, the issue of IFs might be agreed upon by a dynamic process of shared decision-making in which both the patient and professional participate actively [33]. This idea aligns with the suggestion that counselling and consent should not focus strictly on information provision and patients’ individual, rational and autonomous decisions, but should pursue a relational autonomy where patients and professionals reach a decision collaboratively [47]. In such a relational decision-making process, respect for autonomy and beneficence might be expressed in such a way that both values can be respected [48].

Secondly, the mandatory reporting of actionable IFs was partly supported by the assumption that an autonomous patient would agree with disclosure. This suggests that a preference to know is the rational preference because it is well-informed. This idea was also expressed by a Belgian professional who considered a desire to know to be a rationally grounded choice, whereas a desire not to know was usually considered emotionally grounded. However, the association between wanting to know and rationality on the one hand, and not wanting to know and emotion on the other hand, could be challenged. Various emotional reasons are possible for wanting to know IFs, for instance a fearful desire to control life as much as possible. Conversely, as also suggested by professionals who support an opt-out and might value non-maleficence (and autonomy) over beneficence, knowledge can be emotionally disturbing and the rational control over one’s life can necessitate some degree of ignorance [35].

“Rationality sovereignty” [39], which supports the normative standard of wanting to know, is related to the well-known argument of incoherence, which states that ignorance inherently conflicts with autonomy and that autonomous individuals who want to make informed decisions cannot ignore relevant medical information [14, 36]. Likewise, Harris has advocated that patients should be “rational choosers” who base their decisions on “an appropriate level of information” [49]. Whether the argument of incoherence is true or not, it does not apply to the mandatory reporting of IFs. If patients need to be competent and autonomous, they need to be informed about the process of testing, the procedure of ES and its possible consequences, including IFs, *before* testing and not afterwards about actual IFs. The claim that being adequately informed requires the mandatory reporting of IFs confounds the prerequisites of an autonomous decision (i.e. being adequately informed about the genetic testing procedure and possible results) and its possible consequences (i.e. being informed about identified IFs). The only way to validate this claim is to state that ignorance about actionable IFs can impede future autonomous decisions about one’s health and life. However, as stated above, receiving IFs does not absolutely guarantee an enhanced rationality or, as explained below, a better medical and/or psychological outcome.

A last remark should be made on some professionals’ claim that few patients dispute the mandatory reporting of actionable IFs. A central clause of medical paternalism concerns the act of going against patients’ preferences [33]. If most patients seem to value the return of actionable IFs positively, it might be questioned whether the mandatory reporting can actually be classified as a paternalistic intervention. Sandman and Munthe have stated that decisions and interventions are paternalistic whenever they ignore patients’ perspectives, even if they do not explicitly go against patients’ preferences [33]. Even if patients retrospectively approve of professional decisions, their autonomy is partly undermined by being denied some control over the decision-making process [33]. Moreover, this retrospective approval might be the effect of a psychological coping strategy to accept information one cannot unlearn. Nevertheless, from a consequentialist point of view, which also supports paternalism and its beneficent outcomes in general, it might be upheld that the (moral) harm of mandatorily reported IFs is decreased by patients’ retrospective approval of the paternalistic and epistemic intervention.

The mandatory reporting of IFs may certainly result in effective prevention or early treatment of disease. However, it might be questioned whether the soft paternalism regarding actionable IFs is absolutely effective. A minimum requirement for paternalism to be justified is that its benefits outweigh its risks [25]. This claim echoes the screening criteria of Wilson and Jungner and the American Medical Association (AMA) Principles of Medical Ethics, which state that genetic testing is most opportune when it will meaningfully affect a patient’s care [13, 50]. Hence it should be demonstrated that mandatorily reporting actionable IFs will benefit a patient’s health, a claim which is, however, contested sometimes. Knoppers has warned for the “overpromising” of genetic data and, more generally, the pathogenicity, penetrance and expression of variants in asymptomatic persons have been disputed [51]. IFs may vary in reliability and possible use and it should be realised that reporting misinterpreted or uncertain findings might result in unnecessary or harmful follow-ups or interventions [35, 37, 52–55]. Moreover, IFs might cause changes in family, social and professional structures, considerable financial costs, problems regarding insurance or, as already mentioned, emotional harm [35, 36]. For Belgian professionals who support an opt-out possibility, the values of non-maleficence and patient autonomy might take precedence over professional beneficence because of these possible negative consequences of reported IFs. For professionals who reject an opt-out possibility, these potential consequences are, *per contra*, not considered sufficient reasons to outweigh the professional duty of beneficence. If, however, the advantages of reported IFs were surpassed by (possibly underestimated) negative consequences, then the mandatory reporting of IFs invalidates the benefit that paternalism is supposed to provide and violates the professional duty of non-maleficence [55].

This introduces a last topic: the scope of values and, more specifically, the delineation of a patient’s best interest and of a professional’s responsibility and beneficence.

Firstly, this topic was reflected in discussions on reportable IFs. Should only results which might benefit a patient’s medical interest be disclosed? Or should a medical professional also consider results for a patient’s psychological and personal benefit or for the health of his/her family members? Some Belgian professionals referred to a family-wide concept of medical beneficence when arguing for a possible disclosure of IFs regarding a carrier status of a recessive condition, an idea for which international support has increased [7, 56]. Bullock’s context-sensitive evaluation of patients’ best interest further broadens the concept by stating that the disclosure of medical information should be guided by an evaluation of patients’ physical health, their short- and long-term psychological wellbeing and respect for and the facilitation of their (future) autonomy [36]. The delineation of a patient’s best interest is related to the debate on IFs’ actionability and the question of whether these genomic findings should only enable medical interventions or also personally valuable actions, a topic which we have discussed elsewhere [57].

Secondly, questions about the delineation of beneficence were reflected in professionals’ divergent ideas on the spectrum of the professional “clinical gaze”. Whereas some (especially people working in CMGs without an opt-out possibility) advocated a more holistic clinical gaze that is not strictly bound by the diagnostic question, others defended a professional diagnostic focus, in line with patients’ core interest. However, and irrespective of participants’ perspective on the clinical gaze or opting out of IFs, no one has currently recommended the deliberate pursuit of SFs. Belgian professionals showed that they were well-acquainted with the ACMG recommendations on SFs and sometimes they (implicitly) referred to these recommendations to explain or justify their CMG’s policy on IFs. An example concerned a participant’s delineation of the spectrum of reportable IFs (“*Professional bodies have decided that it’s about the actionables. Hence, only cancer and cardiac conditions have been included.*” P11, FG 2). This echo of recommendations on SFs in the discourse about IFs might be partially caused by a limited experience with IFs in clinical ES but it also illustrates these recommendations’ international impact. The intertwinement between the discourse about IFs and the one about SFs can also be discerned in laboratory points to consider, where it is stated that “[i]f a variant on the ACMG list is identified as UF [unsolicited finding] then it should be reported.” [7]. Despite this echoing of recommendations on SFs, there was a consensus among professionals from Belgian CMGs that the active pursuit of SFs currently exceeds the spectrum of beneficence. Underlying arguments were society’s unpreparedness for this practice (an idea associated with genetic illiteracy) and people’s fundamental unwillingness to hear bad news (an idea associated with the duty of non-maleficence). However, these problems could be countered by initiatives that increase people’s genetic literacy and by an absolute opt-out possibility for SFs. Hence the most fundamental argument for the decline of a practice of SFs might be professionals’ statement that this practice is currently an unjust allocation of limited resources. Even though some Belgian professionals were enthusiastic about potentially achieving a “higher level of care” through SFs, this practice was considered currently unfeasible and hence inappropriate. This opinion tallies with the AMA principle that specific care can be denied when it compromises the provision of more fundamental care and with the ethical acknowledgement of a limited professional duty of beneficence because of limited resources and distributive justice [13, 25]. In this sense, Belgian professionals’ perspective conflicts with ACMG members’ evaluation of screening for SFs as standard medical practice and with the suggestion that if diagnostically unrelated information is that valuable, its discovery should not be left to coincidence but actively pursued [1, 5, 52, 58]. Justice, as argued by this last statement, should not be achieved by withholding the opportunity of SFs from patients but by guaranteeing equal access to these results for all patients [52].

In Belgian CMGs, the current lack of resources and the principle of distributive justice may not only justify the current decision not to actively pursue SFs but also the nationwide use of ES-based panel testing (as both practices limit the amount of analysed genes and hence of required resources). This limitation of possible results (including IFs) is supported by international professionals’ questioning of the return and pursuit of IFs and SFs as the most efficient use of limited resources [17, 55, 59, 60]. A filtered analysis minimises (but cannot completely avoid) the chance of IFs and most of the time it is most efficient in terms of diagnostic clinical relevance, it avoids an information overload and it allows clinicians to maximally realise their clinical task [59]. Hence, it is within the boundaries of this panel of available results that concepts of reportable results, opt-out and mandatory disclosure should be considered. It also implies that even the professional duty of geneticists who assign themselves the responsibility of a more holistic clinical gaze is still bound and delineated by the scope of the genetic panel. In other words: for reasons of beneficence, the clinical gaze and the spectrum of professional duty might exceed the diagnostic question but currently, for reasons of distributive justice, this duty does not exceed the scope of the diagnostic panel. Ultimately, this suggests that the value of distributive justice profoundly delineates the scope in which values of autonomy, non-maleficence and beneficence are currently debated. An increase in available resources, decreasing costs of clinical ES-based testing or evolutions in scientific knowledge, societal preferences or people’s genetic literacy may (but not necessarily should) affect the impact of distributive justice, the spectrum of reportable results and the weighing of values in an ethical disclosure policy on IFs and SFs.

## Conclusions

Professionals at Belgian CMGs frequently refer to ethical values for disclosure policies on IFs and SFs. Respect for patient autonomy is invoked to support the disclosure of IFs and opt-out options, non-maleficence to support the delineation of reportable IFs and opt-out options and (the scope of) beneficence to support a mandatory reporting of actionable IFs, the delineation of reportable IFs and a current decline of actively pursued SFs. Additionally, the value of distributive justice largely delineates the scope of reportable results and the spectrum in which ethical values are currently debated. Over the coming years, the spectrum of the ethical debate on IFs and SFs might change and initiatives to improve people’s genetic literacy might affect the legitimacy of a restriction in patient choices on disclosure. Soft paternalism may be acceptable, but the validity of its motives and the plausibility of its beneficent outcome should be continuously verified in the context of scientific, economic and societal evolutions.

This study does not address all aspects of IFs and SFs which require ethical reflection. Topics concerning the informing of family members about IFs, the notification of patients when new information on IFs is available and the implications of patients’ choices for the use of (electronic) health records or patient portals are not included in the scope of this article but definitely require further research. The results of this study emerge from a Belgian context with its specific healthcare structure. However, the way values are weighed in the context of IFs and SFs might be familiar to or instructive for other countries. Therefore, a more international and collective debate on the ethical grounds for a solid (future) policy on IFs and SFs might be highly valuable.

## Data Availability

None of the data generated and analysed during this study are publically available for reasons of personal privacy, but they are available from the corresponding author in response to a reasonable request.

## Abbreviations

ACMG: American College of Medical Genetics and Genomics
AMA: American Medical Association
CCMG: Canadian College of Medical Geneticists
CMG(s): Centre(s) for medical genetics
ES: Exome sequencing
ESHG: European Society of Human Genetics
FG: Focus group
IF(s): Incidental finding(s)
P: Participant
SF(s): Secondary finding(s)
VUS: Variant(s) of uncertain significance
